# Target cell-specific plasticity rules of NMDA receptor-mediated synaptic transmission in the hippocampus

**DOI:** 10.3389/fncel.2023.1068472

**Published:** 2023-04-05

**Authors:** Stefano Lutzu, Karina Alviña, Nagore Puente, Pedro Grandes, Pablo E. Castillo

**Affiliations:** ^1^Dominick P. Purpura Department of Neuroscience, Albert Einstein College of Medicine, Bronx, NY, United States; ^2^Department of Neurosciences, Faculty of Medicine and Nursing, University of the Basque Country UPV/EHU, Leioa, Spain; ^3^Achucarro Basque Center for Neuroscience, Science Park of the University of the Basque Country UPV/EHU, Leioa, Spain; ^4^Department of Psychiatry and Behavioral Sciences, Albert Einstein College of Medicine, Bronx, NY, United States

**Keywords:** CA3, dentate gyrus, mossy cell, synaptic plasticity, calcium signal, two-photon laser scanning microscopy

## Abstract

Long-term potentiation and depression of NMDA receptor-mediated synaptic transmission (NMDAR LTP/LTD) can significantly impact synapse function and information transfer in several brain areas. However, the mechanisms that determine the direction of NMDAR plasticity are poorly understood. Here, using physiologically relevant patterns of presynaptic and postsynaptic burst activities, whole-cell patch clamp recordings, 2-photon laser calcium imaging in acute rat hippocampal slices and immunoelectron microscopy, we tested whether distinct calcium dynamics and group I metabotropic glutamate receptor (I-mGluR) subtypes control the sign of NMDAR plasticity. We found that postsynaptic calcium transients (CaTs) in response to hippocampal MF stimulation were significantly larger during the induction of NMDAR-LTP compared to NMDAR-LTD at the MF-to-CA3 pyramidal cell (MF-CA3) synapse. This difference was abolished by pharmacological blockade of mGluR5 and was significantly reduced by depletion of intracellular calcium stores, whereas blocking mGluR1 had no effect on these CaTs. In addition, we discovered that MF to hilar mossy cell (MF-MC) synapses, which share several structural and functional commonalities with MF-CA3 synapses, also undergoes NMDAR plasticity. To our surprise, however, we found that the postsynaptic distribution of I-mGluR subtypes at these two synapses differ, and the same induction protocol that induces NMDAR-LTD at MF-CA3 synapses, only triggered NMDAR-LTP at MF-MC synapses, despite a comparable calcium dynamics. Thus, postsynaptic calcium dynamics alone cannot predict the sign of NMDAR plasticity, indicating that both postsynaptic calcium rise and the relative contribution of I-mGluR subtypes likely determine the learning rules of NMDAR plasticity.

## Introduction

Long-term changes in synaptic strength are widely regarded as cellular correlates of most forms of learning and memory (Malenka and Bear, 2004; Mayford et al., 2012). The *N*-methyl-D-aspartate receptor (NMDAR) is a critical trigger of long-term potentiation and depression (LTP and LTD) of the fast component of glutamatergic transmission, which is mainly mediated by α-amino-3-hydroxy-5-methyl-4-isoxazolepropionic acid receptors (AMPARs) (Luscher and Malenka, 2012). Like AMPARs, NMDARs are also dynamically regulated and undergo activity-dependent bidirectional long-term plasticity (i.e., NMDAR-LTP and LTD) at many central synapses (Rebola et al., 2010; Hunt and Castillo, 2012). NMDAR plasticity can strongly impact synaptic transmission by changing the threshold of AMPAR plasticity (Rebola et al., 2011; Hunt et al., 2013) and by modulating synaptic integration and neuronal output (Hunt et al., 2013; Banks et al., 2015). However, the molecular mechanisms that control NMDAR plasticity and determine its direction remain unclear. A better understanding of these mechanisms is important since NMDAR dysregulation has been implicated in several brain disorders (Lau and Zukin, 2007), including schizophrenia (Javitt, 2010), autism spectrum disorder (Won et al., 2012), addiction (Borgland et al., 2006) and neurodegenerative diseases such as Alzheimer’s (Liu et al., 2019) and Parkinson’s disease (Gardoni et al., 2010).

The induction of NMDAR plasticity relies on postsynaptic calcium rise, which typically arises from the activation of NMDARs themselves and the recruitment of other calcium sources such as internal calcium stores and calcium influx via voltage-gated calcium channels (Kwon and Castillo, 2008a; Rebola et al., 2008; Harnett et al., 2009; Fernandez de Sevilla and Buno, 2010; Jo et al., 2010; Hunt et al., 2013). The importance of calcium signaling in the bidirectionality of NMDAR plasticity is highlighted by the fact that changing the intracellular calcium buffer capacity with calcium chelators can either block or change the sign of NMDAR plasticity (Harney et al., 2006). In addition, several G protein-coupled receptors (GPCRs) have been implicated in the induction of NMDAR plasticity (MacDonald et al., 2007; Lutzu and Castillo, 2020). Chief among them is the group I metabotropic glutamate receptors (I-mGluRs), which play an essential role in the induction of NMDAR plasticity at several central synapses (Yang et al., 2014; Lutzu and Castillo, 2020). I-mGluRs are G_*q*_-coupled protein receptors whose activation induces calcium release from internal stores (Niswender and Conn, 2010), indicating that mGluRs significantly contribute to the postsynaptic calcium rise that triggers NMDAR plasticity. At the hippocampal mossy fiber-to-CA3 pyramidal cells (MF-CA3) synapse, bidirectional NMDAR plasticity can be elicited by pairing physiologically relevant patterns of presynaptic and postsynaptic burst firing (i.e., burst timing-dependent plasticity), where the order in which pre- and postsynaptic bursts are presented is crucial to establish the direction of NMDAR plasticity (Hunt et al., 2013). Specifically, NMDAR-LTP is triggered if presynaptic burst firing precedes postsynaptic firing, while simply reversing the order of the bursts induces NMDAR-LTD. Both NMDAR-LTP and LTD rely on postsynaptic calcium rise but display different molecular requirements. Whereas NMDAR-LTP requires NMDAR/mGluR5 co-activation and calcium release from intracellular stores, NMDAR-LTD requires NMDAR/mGluR1 co-activation but not calcium release from internal stores (Hunt et al., 2013), suggesting that higher levels of postsynaptic calcium determine the direction of NMDAR plasticity, a possibility never directly tested. Moreover, what additional factors contribute to inducing LTP or LTD of NMDAR-mediated transmission remain unclear.

In the present study, we combined whole-cell patch clamp recordings, 2-photon (2P) calcium imaging and glutamate uncaging in acute rat hippocampal slices, and anatomical analysis via immunoelectron microscopy, to determine the role of postsynaptic calcium rise and I-mGluRs in shaping bidirectional NMDAR plasticity. In addition to the MF-CA3 synapse, we also examined the synapse between MF and hilar mossy cells (MF-MC synapse), which shares several anatomical and functional properties with MF-CA3 synapses (Scharfman, 2016). Our data indicate that NMDAR plasticity is target-cell specific, and that expression of I-mGluRs subtypes combined with postsynaptic calcium dynamics determine the expression and direction of NMDAR plasticity at MF synapses.

## Materials and methods

All experimental procedures described were performed in accordance with the Institutional Animal Care and Use Committee guidelines of the Albert Einstein College of Medicine and the University of the Basque Country and adhered to the guidelines of the USA National Institutes of Health (NIH). Acute transverse slices were prepared from young adult Sprague Dawley rats of both sexes (P17-27). Hippocampi were dissected from the brain and 400 μm thick slices were cut using a vibratome (Leica VT1200S) in ice-cold sucrose solution containing (in mM): 215 sucrose, 20 glucose, 26 NaHCO_3_, 4 MgCl_2_, 4 MgSO_4_, 1.6 NaH_2_PO_4_, 2.5 KCl, and 1 CaCl_2_. Slices were then allowed to recover for ∼20 min at 34°C in 50% sucrose solution and 50% artificial cerebral spinal fluid (ACSF) recording solution containing (in mM): 124 NaCl, 26 NaHCO_3_, 10 glucose, 2.5 KCl, 1 NaH_2_PO_4_, 2.5 CaCl_2_, and 1.3 MgSO_4_. After this recovery period, slices were incubated in ACSF at room temperature (∼25°C) for at least one hour. For electrophysiology and calcium imaging experiments slices were perfused with ACSF bubbled with 95% O_2_ and 5% CO_2_. Unless otherwise stated, during calcium imaging experiments ACSF was supplemented with the GABA_*A*_ receptor antagonist picrotoxin (30 μM) to block fast synaptic inhibition, and 0.5–1 μM LY303070, a non-competitive selective AMPAR antagonist, to minimize the activation of the CA3 recurrent network (Kwon and Castillo, 2008b).

### Electrophysiology

Slices were transferred to a recording chamber perfused with ACSF bubbled with 95% O_2_ and 5% CO_2_ and kept at room temperature (∼25°C). The flow rate was adjusted to 2 ml/min. Visualized whole-cell patch clamp recordings from CA3 pyramidal neurons and MCs were performed using borosilicate glass pipettes (3–6 MΩ tip resistance) filled with a potassium-based internal solution containing: 135 mM KMeSO_3_, 10 mM HEPES, 4 mM MgCl_2_, 4 mM Na_2_ATP, 0.4 mM NaGTP and 10 mM Na^+^ creatine phosphate; 290 mOsm; pH 7.3 corrected with KOH. For calcium imaging, the low-affinity calcium indicator Fluo5f (300 μM) and the morphological fluorescent indicator Alexa 594 (20 μM) were added to the internal solution.

**FIGURE 1:**
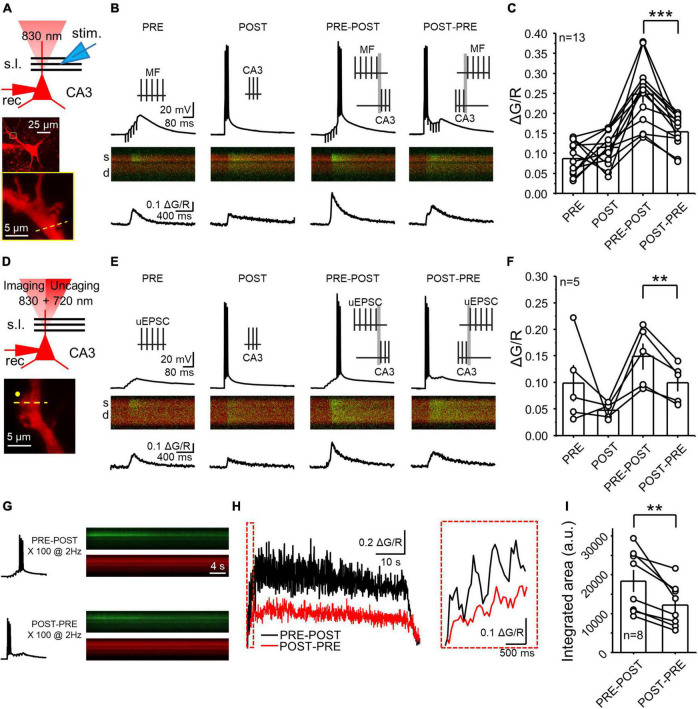
NMDAR LTP was associated with a larger postsynaptic calcium rise than NMDAR LTD at the MF-CA3 synapse. **(A)** Experimental recording configuration (top). Simultaneous excitation of Alexa 594 and Fluo-5f loaded via a patch pipette was performed with a 2P laser tuned at 830 nm. Representative CA3 pyramidal cell loaded with Alexa 594 (middle) and inset showing magnification of the imaged postsynaptic TE (yellow box). High magnification image showing the scanning line (yellow dashed line) on the TE (bottom). s.l.: stratum lucidum; θ stim: theta glass stimulus pipette; rec: recording pipette. **(B)** Electrophysiological recordings and calcium responses obtained from a CA3 pyramidal neuron. Stimulation patterns employed (top): PRE (MF electrical stimulation – 5 pulses at 50 Hz); POST (CA3 pyramidal cell action potentials – 3 spikes at 100 Hz); PRE-POST (delay 10 ms between bursts); and POST-PRE (delay 10 ms between bursts). Fluorescence profile (middle) of the green signal (Fluo5f) superimposed with the red signal (morphological dye Alexa 594). S: spine; d: dendrite. Fluorescence quantification reported as ΔG/R over time (bottom). **(C)** Summary of mean peak ΔG/R across all patterns of stimulation. Here and in all figures, circles linked by a line represent individual experiments performed in the same cell. **(D)** Experimental configuration used to perform electrophysiology, calcium imaging and glutamate uncaging (top). Excitation wavelengths employed were 830 nm (for imaging) and 720 nm (for glutamate uncaging). High magnification image showing the position of the uncaging laser beam (yellow circle) the scanning line (yellow dashed line) on the TE (bottom). **(E,F)** Same as panels **(B,C)** but employing glutamate uncaging instead of MF stimulation. **(G)** Single electrophysiological response (left) and fluorescence profile during full induction protocol for NMDAR LTP and LTD (right). **(H)** Calcium responses of the experiment showed in panel **(G)** expressed as ΔG/R over time. Boxed area shows a magnification of ΔG/R during the first 4 pairings. **(I)** Summary of ΔG/R change during the full induction protocols expressed as integrated area under the curve. Data are presented as mean ± SEM. **p < 0.01, ***p < 0.001.

Data were collected using a MultiClamp 700B amplifier (Molecular Devices, San Jose, CA). Signals were filtered at 2.4 kHz and acquired at 5 kHz with custom software written in IgorPro. CA3 pyramidal cells and MCs were initially voltage clamped at ∼–65 mV and the internal solution was allowed to equilibrate for at least 30 min. During this time window, we checked for the presence and stability of MF-derived AMPAR excitatory postsynaptic currents (EPSCs) using an extracellular bipolar theta glass stimulator which was placed in the stratum lucidum for MF-CA3 recordings, or in the subgranular zone of the dentate gyrus for MF-MC recordings. In both cases, the stimulating pipette was placed ∼50–100 μm from the patched cell. MF-evoked responses were elicited with a short (80 μs) current square pulse. Stimulation intensity ranged from 20 to 50 μA. For both types of recordings, MF-derived responses were identified according to the high presynaptic facilitation (P2/P1 > 2), fast rise time (<2 ms) and high sensitivity to the mGluR2/3 agonist DCG-IV (1 μM) (>80% reduction of the synaptic response). After the initial ∼30 min in voltage clamp, recordings were switched to current clamp mode while keeping the membrane resting potential at −70 mV. The resting potential was adjusted to more hyperpolarized potentials (∼–75 mV) if synaptically-driven spikes were evoked during MF burst stimulation. The induction protocols for NMDAR-LTP and LTD were designed as previously described (Hunt et al., 2013). Briefly, NMDAR-LTP and LTD induction protocols consisted of pairings of presynaptic MF bursts (PRE) (5 pulses at 50 Hz) with postsynaptic bursts of CA3 pyramidal cells (POST) (3 action potentials at 100 Hz) which were evoked with brief (2–3 ms) injections of current (1–1.5 nA) in the postsynaptic cell. The NMDAR-LTP protocol consisted of 100 PRE-POST pairings (i.e., PRE leading POST stimulation) with a 10 ms delay in between, delivered at a 2 Hz inter-pairing interval. NMDAR-LTD was induced by reversing the burst order (i.e., POST-PRE) with the same 10 ms delay. All calcium measurements were performed using single pairings except those in Figures 1G–I, where the full induction protocol with one hundred pairings was used, and in Figure 3 where only PRE stimulation patterns were used. Recordings showing access resistance >25 MΩ or changes exceeding 15% were discarded. To measure DHPG-induced currents we patched CA1 pyramidal cells in voltage-clamp mode at −60 mV in presence of 10 μM NBQX, 100 μM picrotoxin, and 3 μM CGP and 25 μM D-APV (to block AMPA/Kainate, GABA_*A*_, GABA_*B*_, and NMDA receptors, respectively). The DHPG-mediated change in holding current was measured by subtracting the average value of the holding current during the last 3 min of a 10-min DHPG application to the average value of the baseline period (ΔI Holding).

For the induction of NMDAR plasticity in MCs, visualized whole-cell patch clamp recordings from MCs were performed using a K^+^-based internal solution containing: 135 mM KMeSO_4_, 5 mM KCl, 1 mM CaCl_2_, 5 mM NaOH, 10 mM HEPES, 5 mM MgATP, 0.4 mM Na_3_GTP, 5 mM EGTA, 10 mM D-glucose, pH 7.2 (280–290 mOsm). MCs were identified using previously established criteria (Larimer and Strowbridge, 2008): elevated spontaneous synaptic activity, little to no afterhyperpolarization and non-burst firing patterns. These criteria were validated by patch-loading cells with Alexa 594, which allowed us to image the cell and confirm the presence of TEs on MCs (Scharfman, 2016). To enhance response detectability, MF-derived NMDAR EPSCs were elicited with 2 pulses at 200 Hz in the presence of 10 μM NBQX, 100 μM picrotoxin, and 3 μM CGP (to block AMPA/Kainate, GABA_*A*_, and GABA_*B*_ receptors respectively).

### 2-photon calcium imaging and glutamate uncaging

Calcium imaging and glutamate uncaging measurements were performed using an Ultima 2-photon laser scanning microscope (Bruker Corporation, Billerica, MA) equipped with both an Insight Ti:Sapphire laser and a MaiTai laser (Spectra Physics, MKS Instruments, Inc., Andover, MA). The excitation wavelength for imaging and uncaging were set at 830 and 720 nm, respectively. For calcium imaging, fluorescence across the region of interest was detected in line scan mode at high frequency (500 Hz) using Prairie View 5.4 software (Bruker Corporation). Postsynaptic CaTs were estimated by calculating the ΔG/R ratio, which represents the stimulation-induced change in fluorescence from the baseline of the green channel (Fluo-5f signal), normalized by the fluorescence of the Red channel (Alexa-594 signal). PRE, POST, PRE-POST, and POST-PRE CaTs were quantified as the average peak ΔG/R of at least 5 trials. All trials were acquired with at least 1 min delay. To assess the effect of drug application on PRE CaTs, the peak ΔG/R after drugs wash-in (average of at least 5 traces acquired during a 5–10 min long baseline) was normalized to the average of the CaTs acquired during the baseline period (average of at least 5 traces acquired during the last 15 min of 30 min drug wash-in). Only trials in which synaptic MF stimulation did not induce spikes were analyzed. To avoid potential priming effects due to the PRE-POST, or POST-PRE stimulation, we delivered these protocols in an interleaved fashion in different cells.

For glutamate uncaging experiments 2.5 mM MNI-caged-L-glutamate was added to the ACSF and used to perfuse the slice. The uncaging laser was parked ∼1 μm away from the head of the target dendritic spine and 5 uncaging pulses (0.5 ms duration, at 50 Hz) were used to mimic glutamate release from MFs with the same frequency used for electrical stimulation of MF. The uncaging laser power was adjusted to detect a measurable calcium signal and avoid postsynaptic spikes during the short burst. To compare the difference between 100 PRE-POST and POST-PRE pairings, measurements were performed in the same cell. To avoid any potential confounding effect due to changes in NMDAR function, the two protocols were elicited within 5 min from each other, before the onset of the expression of NMDAR plasticity (Hunt et al., 2013). Moreover, the two protocols were elicited by alternating the order in different cells to account for potential priming effects between protocols.

### Double pre-embedding immunoelectron microscopy

Long Evans rats (*n* = 3) were deeply anesthetized by intraperitoneal injection of ketamine/xylazine (80/10 mg/kg body weight) and transcardially perfused at room temperature (RT, 20–25°C) with phosphate buffered saline (0.1 M PBS, pH 7.4) for 20 s, followed by the fixative solution (4% formaldehyde freshly depolymerized from paraformaldehyde, 0.2% picric acid and 0.1% glutaraldehyde) in PBS (0.1 M, pH 7.4) for 10–15 min. Brains were removed from the skull and post-fixed in the fixative solution for about 1 week at 4°C and stored at 4°C in 1:10 diluted fixative solution until use.

The procedure has been previously described (Puente et al., 2019). Briefly, coronal hippocampal vibrosections were cut at 50 μm and collected in phosphate buffer (0.1 M PB, pH 7.4) with 0.1% sodium azide at RT. They were transferred and pre-incubated in a blocking solution of 10% bovine serum albumin (BSA), 0.1% sodium azide and 0.02% saponin prepared in Tris-hydrogen chloride buffered saline 13 (TBS), pH 7.4 for 30 min at RT. Then, the hippocampal sections were incubated with the primary rabbit polyclonal anti-mGluR1b receptor antibody (2 mg/ml, mGluR1b–Rb–Af250, Frontier Institute Co. Ltd, Ishikari, Hokkaido, Japan, RRID:AB_2616586) or guinea pig polyclonal anti-mGluR5 antibody (2 mg/ml, mGluR5b–GP–Af270–1, Frontier Institute Co. Ltd, Ishikari, Hokkaido, Japan, RRID:AB_2571804) diluted in 10% BSA/TBS containing 0.1% sodium azide and 0.004% saponin on a shaker for 2 days at 4°C. Both antibodies were extensively characterized previously, and the characteristic labeling in wildtype tissue disappeared in the brains of mGluR1 and mGluR5 knock–out mice (Ferraguti et al., 1998; Mateos et al., 1998; Tanaka et al., 2000; Uchigashima et al., 2007; Ohtani et al., 2014). Tissue was incubated after several washes in 1% BSA/TBS with the biotinylated secondary antibody (1:200 goat biotinylated anti-rabbit, Cat# BA-1000, Vector Labs, Burlingame, CA, USA; RRID:AB_2313606 or 1:200 donkey biotinylated anti-guinea pig Cat#, 706-065-148, Jackson ImmunoResearch Labs, West Grove, PA, USA; RRID:AB_2340451) in 1% BSA/TBS with 0.004% saponin for 3 h at RT. The sections were then washed in 1% BSA/TBS overnight on a shaker at 4°C and incubated with either 1.4 nm gold-labeled goat anti-rabbit IgG antibody (Fab’ fragment, 1:100, Cat # #2004, Nanoprobes Inc., Yaphank, NY, USA) or 1.4 nm gold-labeled goat anti-guinea pig IgG antibody (Fab’ fragment, 1:100, Cat # #2055, Nanoprobes Inc., Yaphank, NY, USA) in 1% BSA/TBS with 0.004% saponin on a shaker for 3 h at RT, for mGluR1b or mGluR5 labeling, respectively. Next, the sections were washed in 1% BSA/TBS and subsequently incubated in the avidin-biotin complex (1:50; PK-7100, Vector Labs, Burlingame, CA, USA; RRID:AB_2336827) diluted in the wash solution for 1.5 h. After washing in 1% BSA/TBS overnight at 4°C, tissue was post-fixed with 1% glutaraldehyde in TBS for 10 min and washed in double-distilled water. Afterward, the gold particles were silver intensified with an HQ Silver kit (Cat#2012; Nanoprobes Inc., Yaphank, NY, USA) for about 12 min in the dark, washed in 0.1 M PB (pH 7.4) and subsequently incubated in 0.05% DAB (MilliporeSigma, Cat#D5637; RRID:AB_2336819) and 0.01% hydrogen peroxide prepared in 0.1 M PB for 3 min. Finally, the sections were osmicated (1% osmium tetroxide, Electron Microscopy Sciences, Cat#19150) in 0.1 M PB pH 7.4 for 20 min, washed in 0.1 M PB (pH 7.4), dehydrated in graded alcohols (50%–100%) to propylene oxide and plastic-embedded in Epon resin 812. 50–60 nm-ultrathin sections were cut with a diamond knife (Diatome USA), collected on nickel mesh grids, stained with 2.5% lead citrate, and examined with a JEOL JEM 1400 Plus electron microscope. Tissue samples were imaged using a digital camera (sCMOS).

The double immunocytochemical method was repeated three times on the sections obtained from each of the three brains studied. Immunoperoxidase and immunogold-labeling were visualized on the hippocampal sections with a light microscope and portions of the CA3 stratum lucidum and the dentate hilus with good and consistent mGluR1b and mGluR5 receptors immunolabeling were identified and trimmed down for ultrathin sectioning. Three to four semi-thin sections (1 μm-thick) were then cut with a histo-diamond knife (Diatome USA) and stained with 1% toluidine blue. To further standardize the conditions, only the first 20 ultrathin sections (50–60 nm thick) were cut, collected onto the grids and photographed. The electron micrographs were taken at 8,000× with a Digital Morada Camera from Olympus (Hamburg, Germany). Sampling was always carefully and accurately carried out in the same way for the brains studied and it was blinded to experimenters during mGluRs quantification.

### Data analysis and statistics

*Electrophysiology and calcium imaging.* Graphs and statistical analysis were performed using OriginPro (Origin Laboratory, Northampton, MA). The normality of data distribution was assessed with the Shapiro–Wilk test. Data with a normal distribution were analyzed using a parametric test (One-Sample t Test for unpaired data and Paired t Test for paired data), whereas data that did not display a normal distribution were analyzed with a non-parametric test (Mann–Whitney). For multiple comparisons, we employed a One-Way ANOVA with a Bonferroni *post hoc* analysis for mean comparison. Statistical significance was set at *P* < 0.05.

*Immunoelectron microscopy.* The double immunocytochemical method was repeated three times on the sections obtained from each of the three brains that were analyzed. In particular, a total of 181 dendritic thorny excrescences (TEs) of pyramidal neurons contacted by MF terminals in CA3 stratum lucidum (area studied: 1406.1 μm^2^) and 153 mossy cell TEs with MF synapses in dentate hilus (area studied: 1406.1 μm^2^) were analyzed. Positive mGluR1b and/or mGluR5 TEs were identified by the presence of DAB immunodeposits and/or by the presence of at least one gold particle within 30 nm of the postsynaptic membrane. Collecting data of mGluR1b/mGluR5 positive TEs obtained for each antibody in CA3 and in the dentate hilus were pooled. The percentage of mGluR1b and/or mGluR5 positive TEs was analyzed and displayed using a statistical software package (GraphPad Prism 8, GraphPad Software Inc, San Diego, CA, USA; RRID:SCR_002798). Data are presented as mean ± SEM. The data were pooled since three analyzed samples did not differ in mGluRs labeling (Kolmogorov Smirnov test, *P* > 0.19). Then, the ratio of the total mGluR5 versus total mGluR1b in TEs of CA3 and dentate hilus was compared. The normality test (Kolmogorov–Smirnov normality test) was applied before statistical tests and subsequently, data were analyzed using a parametric unpaired t Test.

### Drugs and chemicals

All chemicals and drugs used were purchased from MilliporeSigma (St. Louis, MO, USA) except D-APV, NBQX, CGP-55845, DCG-IV, MNI-caged-L-glutamate and CPA which were obtained from Tocris Bioscience (Minneapolis, MN, USA), LY 303070 which was obtained from ABX advanced biochemical compounds (Radeberg, Germany), and Fluo5f and Alexa which were purchased from Invitrogen (Carlsbad, CA, USA).

## Results

### The magnitude of postsynaptic calcium rise during NMDAR-LTP and LTD induction differs

To assess the postsynaptic calcium signals elicited by distinct patterns of activity that induce NMDAR-LTP and NMDAR-LTD at MF-CA3 synapses, we combined whole-cell patch clamp recordings and 2P calcium imaging in acute rat hippocampal slices. We patch-loaded CA3 pyramidal cells with a potassium-based internal solution containing the low-affinity calcium indicator Fluo5f (300 μM), and Alexa 594 (20 μM) which was used as a morphological indicator. We performed recordings in current-clamp configuration in the presence of picrotoxin (30 μM) to block GABA_*A*_-mediated transmission, and a low concentration of LY303070 (0.5–1 μM) to minimize AMPAR-mediated synaptic potentials arising from the recurrent associational-commissural network (Kwon and Castillo, 2008b). Under these recording conditions, presynaptic MF stimulation (e.g., 5 stimuli, 50 Hz) evoked subthreshold EPSPs that were associated with calcium transients (CaTs) in the target postsynaptic CA3 spines thorny excrescences (TEs) (Figures 1A–C; ΔG/R PRE = 0.09 ± 0.01) and, as expected, direct activation of CA3 pyramidal neurons (3 action potentials, 100 Hz) also induced CaTs in TEs (Figures 1A–C; POST = 0.12 ± 0.01). As previously reported, activation of postsynaptic glutamate receptors, calcium release from the internal stores, and activation of voltage-gated calcium channels likely accounts for these CaTs (Kapur et al., 1998, 2001; Reid et al., 2001). We next analyzed CaTs elicited by single PRE-POST and POST-PRE pairings which are known to trigger burst timing-dependent NMDAR-LTP and LTD, respectively (Hunt et al., 2013). Single pairings provide an estimation of the postsynaptic calcium changes occurring during the full induction protocol of plasticity (Koester and Sakmann, 1998; Nevian and Sakmann, 2004; Mishra et al., 2016). We found that PRE-POST pairings elicited larger CaTs than POST-PRE pairings (Figures 1B, C, ΔG/R PRE-POST = 0.25 ± 0.02; POST-PRE = 0.16 ± 0.01; *n* = 13; PRE-POST vs POST-PRE: paired t Test *p* = 0.00026), indicating that the induction of NMDAR-LTP is likely associated with larger postsynaptic calcium rise than NMDAR-LTD.

Extracellular MF stimulation could recruit extrinsic neuromodulatory fibers and trigger the release of modulators from the MF boutons, both of which could potentially affect postsynaptic CaTs. To directly address this issue, we employed 2P glutamate uncaging and tested whether glutamate alone could mimic the different CaTs following PRE-POST and POST-PRE pairing protocols with MF extracellular electrical stimulation. We found that 2P glutamate uncaging ∼1 μm from TEs (Figure 1D) elicited remarkably similar PRE-POST and POST-PRE CaTs as observed with electrical stimulation (Figures 1E,F, ΔG/R uncaging PRE = 0.09 ± 0.03; POST = 0.05 ± 0.01; PRE-POST = 0.15 ± 0.02; POST-PRE: 0.10 ± 0.02; PRE-POST vs POST-PRE: paired t Test *p* = 0.0099). These results indicate that the different calcium signals elicited by PRE-POST and POST-PRE pairing protocols are due to the direct activation of postsynaptic glutamate receptors.

To determine whether the difference in calcium signals observed with single pairings was preserved during the full induction protocol, which comprises 100 PRE-POST (NMDAR-LTP) or POST-PRE (NMDAR-LTD) pairings delivered at 2 Hz respectively (Hunt et al., 2013), we measured CaTs while delivering these protocols in the same cell (Figure 1G). An equal number of cells received a single LTP or LTD protocol first, and the second protocol was delivered 5 min after the first one (see Methods). We found that the NMDAR-LTP induction protocol elicited larger CaTs compared to the NMDAR-LTD induction protocol (Figures 1H,I). To quantify this difference, we calculated the integrated area under the curve in arbitrary units (a.u.) (Figures 1H, I, PRE-POST = 18,414.30 ± 2,833.90 a.u.; POST-PRE = 12,229.05 ± 1,964.84 a.u.; PRE-POST vs POST-PRE: *n* = 8, paired t Test, *p* = 0.00529). Altogether, our results indicate that NMDAR-LTP induction is associated with a larger calcium rise than NMDAR-LTD induction and that postsynaptic glutamate receptors most likely account for this difference.

### mGluR1 and mGluR5 contribute differently to postsynaptic calcium rise evoked by PRE-POST and POST-PRE patterns of activity

**FIGURE 2:**
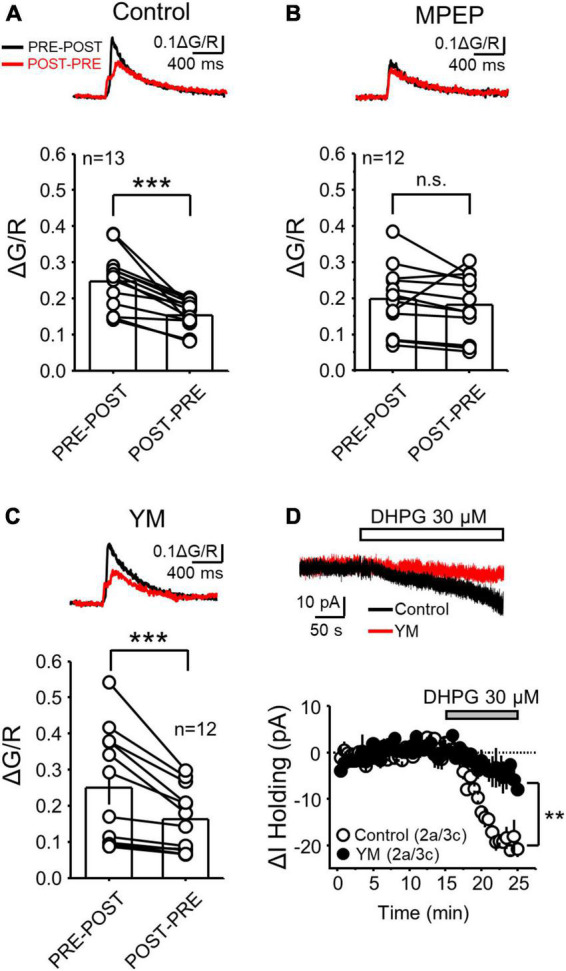
mGluR5 but not mGluR1 activation was critical for the difference in postsynaptic calcium rise during PRE-POST and POST-PRE at MF-CA3 synapses. **(A)** Representative traces of PRE-POST and POST-PRE fluorescence profiles in control conditions (top) and summary plots showing the average ΔG/R measured during PRE-POST and POST-PRE patterns (same dataset from Figure 1C) (bottom). **(B,C)** Same experimental design as in A but in presence of the mGluR5 antagonist MPEP (4 μM) **(B)** and the mGluR1 antagonist YM (1 μM) **(C)**. **(D)** Representative inward current mediated by bath application of the group I mGluR agonist DHPG (30 μM) in control and during YM co-application (top). Time-course summary plot of the DHPG-mediated change in holding current (ΔI Holding) recorded from voltage-clamped CA3 pyramidal cells under control conditions and in the presence of YM. Here and in all figures: a, number of animals and c, number of cells. Data are presented as mean ± SEM). **p < 0.01, ***p < 0.001, n.s. not significant.

At the MF-CA3 synapse, mGluR5 and mGluR1 are required for burst timing-induced NMDAR-LTP and LTD, respectively (Hunt et al., 2013). Because G_*q*_-coupled receptors activate the phosphoinositide pathway that mediates calcium release from calcium stores (Niswender and Conn, 2010), we hypothesized that co-activation of NMDAR and mGluR1 or mGluR5 determines the magnitude of the postsynaptic calcium that triggers MF-CA3 NMDAR-LTP and LTD. To test this hypothesis, we delivered the PRE-POST and POST-PRE stimulation pairings in the presence of mGluR1 or mGluR5 selective antagonists. As previously reported, mGluR1 and mGluR5 antagonism has no significant effect on basal MF-CA3 synaptic transmission (Hunt et al., 2013). In comparison to control conditions (Figure 2A, Data replotted from Figure 1C for comparison reasons), bath application of the non-competitive mGluR5 antagonist MPEP (4 μM) abolished the difference between PRE-POST and POST-PRE CaTs (Figure 2B, MPEP ΔG/R PRE-POST = 0.20 ± 0.03; POST-PRE = 0.18 ± 0.03; *n* = 12, paired t Test *p* = 0.347). Conversely, blockade of mGluR1 with the non-competitive antagonist YM298198 (1 μM) did not alter the difference between PRE-POST and POST-PRE observed in control conditions (Figure 2C, YM ΔG/R PRE-POST = 0.25 ± 0.04; POST-PRE = 0.16 ± 0.04; *n* = 12, paired t Test: *p* = 0.0025). As positive control that YM was efficiently blocking mGluR1, in a separate set of experiments we found that the inward current mediated by the I-mGluR agonist DHPG (30 μM) in CA1 pyramidal neurons was significantly reduced by YM co-application, as previously reported (Mannaioni et al., 2001; Rae and Irving, 2004) (Figure 2D, Control: −19.49 ± 1.28 pA, *n* = 3; YM: −4.46 ± 2.63 pA, *n* = 3; two sample t-test *p* = 0.0068). These results indicate that mGluR1 and mGluR5 contribute differently to postsynaptic calcium rise at MF-CA3 synapses, consistent with their distinct role in NMDAR-LTP and LTD.

### Glutamate release during MF burst activity engages diverse postsynaptic calcium sources

**FIGURE 3:**
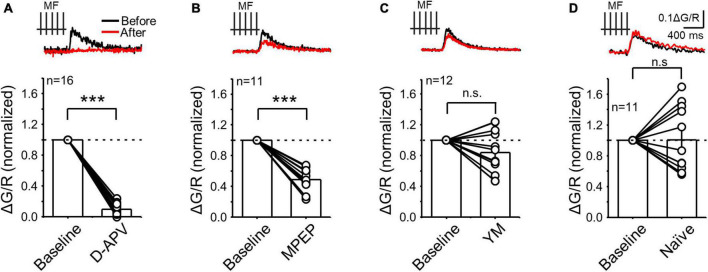
NMDAR and mGluR5 but not mGluR1 contributed to postsynaptic calcium rise evoked during presynaptic MF burst activity. **(A)** Calcium signals induced by presynaptic stimulation (PRE) before and after bath application of the NMDAR antagonist D-APV (25 μM). Representative traces (top) and summary plot of the averaged peak ΔG/R before (normalized to baseline) and after drug application. **(B–D)** Same experimental design as in panel **(A)** but following application of the mGluR5 antagonist MPEP (4 μM) **(B)**, the mGluR1 antagonist YM (1 μM) and in naïve conditions (i.e., calcium signals monitored during 15–30 min in the absence of drug applications) to evaluate stability. Data are presented as mean ± SEM. ***p < 0.001, n.s. not significant.

Previous studies at the MF-CA3 synapse reported that glutamate triggers postsynaptic calcium rise via activation of ionotropic receptors and/or via mGluR-mediated calcium release from intracellular stores (Jaffe and Brown, 1997; Kapur et al., 2001; Reid et al., 2001). Of note, calcium signals in these studies were elicited by non-physiological tetanic stimulation (e.g., 100 Hz, 1 s) (Jaffe and Brown, 1997), single stimulation in organotypic slice cultures (Reid et al., 2001), or were analyzed using a low-spatial resolution imaging approach (Kapur et al., 2001). We therefore tested the contribution of ionotropic glutamate receptors and mGluRs in generating postsynaptic calcium signals elicited by physiologically relevant MF burst stimulation (PRE only) and monitored using 2P calcium imaging in acute rat hippocampal slices. After obtaining baseline CaTs in response to a short burst of MF stimulation (5 pulses, 50 Hz), we blocked NMDARs with the competitive antagonist D-APV (25 μM). This manipulation nearly abolished postsynaptic CaTs (Figure 3A, PRE + D-APV = 0.10 ± 0.02 ΔG/R normalized to control; *n* = 16, paired t Test: *p* = 0.000005), indicating that NMDARs are robustly engaged during MF burst activity and mediate most of the postsynaptic calcium rise despite being expressed at a lower level than AMPARs (Takumi et al., 1999).

In separate experiments, the mGluR5 antagonist MPEP (4 μM) significantly reduced postsynaptic CaTs (Figure 3B, PRE + MPEP = 0.49 ± 0.04 ΔG/R normalized to control, *n* = 11, paired t Test: *p* = 0.000041), suggesting that MF burst activity increases postsynaptic calcium via mGluR5 and NMDAR co-activation. In contrast, mGluR1 antagonism with YM (1 μM) had no significant effect on CaTs (Figure 3C, PRE + YM = 0.84 ± 0.08 ΔG/R normalized to control, *n* = 12. paired t Test: *p* = 0.139). Likewise, no significant changes were observed in naïve slices during 15–30 min, i.e., after no drug application (Figure 3D, PRE – 15–30 min = 1.02 ± 0.13 normalized ΔG/R, *n* = 11, Wilcoxon signed ranks test: *p* = 0.83). Overall, our results strongly suggest that mGluR5 and mGluR1 engage different intracellular cascades implicated in MF-CA3 postsynaptic calcium rise.

### Intracellular calcium stores determine the calcium rise magnitude elicited by NMDAR-LTP and LTD induction

**FIGURE 4:**
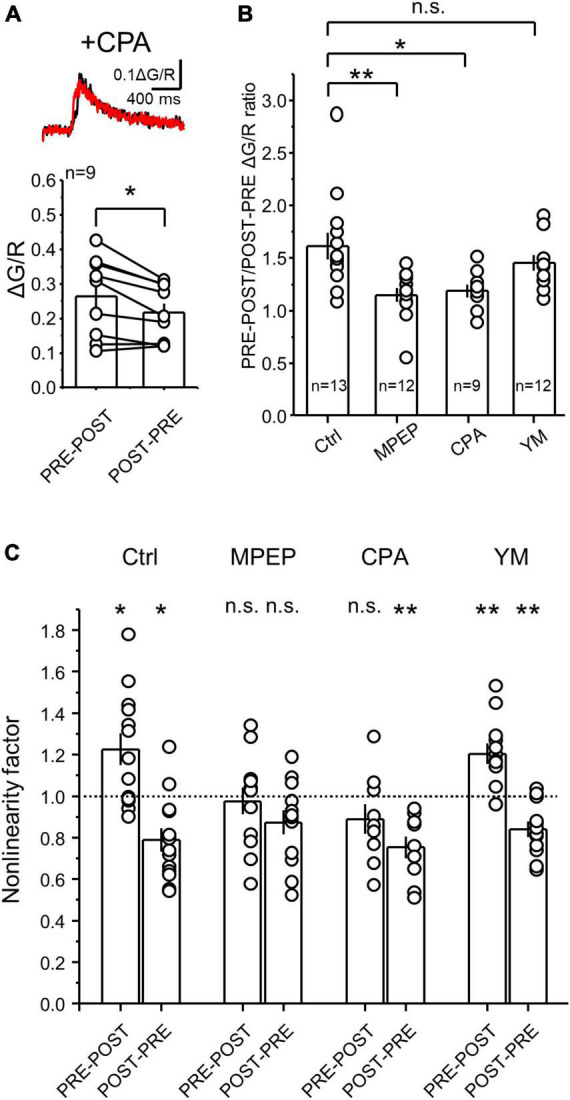
Calcium depletion from internal stores significantly reduced the difference between PRE-POST and POST-PRE CaTs at MF-CA3 synapses. **(A)** Representative CaTs induced by PRE-POST and POST-PRE stimulation in the presence of the SERCA pump blocker cyclopiazonic acid (CPA) (top). Brain slices were incubated for 30 min in 30 μM CPA which was also continuously perfused. Summary plot showing the effect of CPA on peak ΔG/R measured during PRE-POST and POST-PRE stimulation (bottom). **(B)** PRE-POST and POST-PRE CaT ratio under different experimental conditions: Ctrl (no drugs), 4 μM MPEP, 30 μM CPA and 1 μM YM. One Way ANOVA; *F* = 5.86458; *p* = 0.00194; DF = 3; Ctrl vs MPEP, *p* = 0.0036; Ctrl vs CPA, *p* = 0.0198; Ctrl vs YM, *p* = 0.136; Bonferroni *post hoc* analysis. **(C)** Non-linear summation of postsynaptic calcium signals under different experimental conditions. Non-linearity was measured as the ratio between the measured PRE-POST or POST-PRE CaTs and the arithmetical sum of the PRE and POST components alone. Analysis was performed on data shown in Figures 2B–D, 4A. All data points were compared against a non-linearity factor of 1 using One Sample t Test. Data are presented as mean ± SEM. **p < 0.01, *p < 0.05, n.s. not significant.

The postsynaptic calcium rise mediated by mGluRs is likely due to their positive coupling to calcium release from internal stores, such as the endoplasmic reticulum (ER) (Kapur et al., 2001; Reid et al., 2001). Therefore, we predicted that depletion of calcium from the ER should reduce the difference between single PRE-POST and POST-PRE pairings. Indeed, this difference was significantly reduced in hippocampal slices incubated for at least 30 minutes with the SERCA pump blocker cyclopiazonic acid (CPA, 30 μM) which depletes the calcium content from the ER (Figure 4A, ΔG/R PRE-POST = 0.26 ± 0.04; ΔG/R POST-PRE = 0.22 ± 0.03; PRE-POST vs POST-PRE: *n* = 9, Paired t Test *p* = 0.01116; see Figure 2A). To better quantify the effect of CPA, we calculated the PRE-POST/POST-PRE ratio of CaTs, which allows the comparison across different recording conditions. This analysis revealed that mGluR5 antagonism and/or CPA-mediated depletion of calcium stores, but not mGluR1 antagonism, abolished the difference between PRE-POST and POST-PRE CaTs (Figure 4B, PRE-POST/POST-PRE; Ctrl = 1.62 ± 0.13, *n* = 13; MPEP = 1.15 ± 0.07 *n* = 12; CPA = 1.20 ± 0.06, *n* = 9; YM = 1.45 ± 0.07, *n* = 12; One Way ANOVA, *F* = 5.865; *p* = 0.00194; DF = 3.; Ctrl vs MPEP *p* = 0.0036; Ctrl vs CPA *p* = 0.0198; Ctrl vs YM *p* = 0.136; Bonferroni *post hoc* test). These results indicate that the PRE-POST, but not the POST-PRE protocol engages mGluR5 signaling and calcium release from intracellular stores, two key determinants of the distinct CaTs between NMDAR-LTP and LTD.

Pairing of presynaptic and postsynaptic action potentials with short delays can elicit non-linear summation of postsynaptic calcium signals (Yuste and Denk, 1995; Koester and Sakmann, 1998), which is thought to be important for bidirectional plasticity of AMPAR-mediated transmission (Nevian and Sakmann, 2004, 2006). To test for potential non-linear summation of postsynaptic calcium signals generated by MF-CA3 PRE-POST and POST-PRE pairing under different conditions, we performed a non-linearity analysis by calculating a non-linearity factor (NLF), which is the ratio between the *measured* PRE + POST summation (i.e., PRE-POST CaTs peak) and the *predicted* PRE + POST summation (i.e., the arithmetical sum between PRE and POST peak CaTs) (Nevian and Sakmann, 2004). We found that under control conditions, the PRE-POST pairing is supralinear (i.e., NLF > 1), while the POST-PRE coupling is significantly sublinear (i.e., NLF < 1) (Figure 4C). Consistent with a critical role of mGluR5 and their downstream targets calcium stores in the generation of different CaTs levels between PRE-POST and POST-PRE, both MPEP and CPA abolished the supralinear summation of PRE and POST CaTs. In contrast, the blockade of mGluR1 with YM, did not affect the non-linearity factor of the PRE-POST and POST-PRE pairings, (Figure 4C, NLF Ctrl PRE-POST = 1.22 ± 0.07, *n* = 13, *p* = 0.01; Ctrl POST-PRE = 0.78 ± 0.05, *n* = 13, *p* = 0.003; MPEP PRE-POST = 0.97 ± 0.06, *n* = 12, *p* = 0.72; MPEP POST-PRE = 0.87 ± 0.05, *n* = 12, *p* = 0.054; YM PRE-POST = 1.20 ± 0.05, *n* = 12, *p* = 0.002; YM POST-PRE = 0.84 ± 0.03, *n* = 12, *p* = 0.001; CPA PRE-POST = 0.90 ± 0.07, *n* = 9, *p* = 0.16; CPA POST-PRE = 0.75 ± 0.05, *n* = 9, *p* = 0.001; One Sample t Test, all comparisons were against the value NLF = 1; Ctrl PRE-POST vs POST-PRE, *p* = 0.00001; MPEP PRE-POST vs POST-PRE, *p* = 0.91; CPA PRE-POST vs POST-PRE, *p* = 0.846; YM PRE-POST vs POST-PRE, *p* = 0.0011; One-Way ANOVA, *F* = 9.19144; DF = 7; Bonferroni *post hoc* test). Taken together these results suggest that mGluR5, but not mGluR1, activation and calcium release from the internal stores are critical for the larger calcium rise associated with the induction of NMDAR-LTP.

### NMDAR plasticity at the MF-to-Hilar mossy cell synapse

**FIGURE 5:**
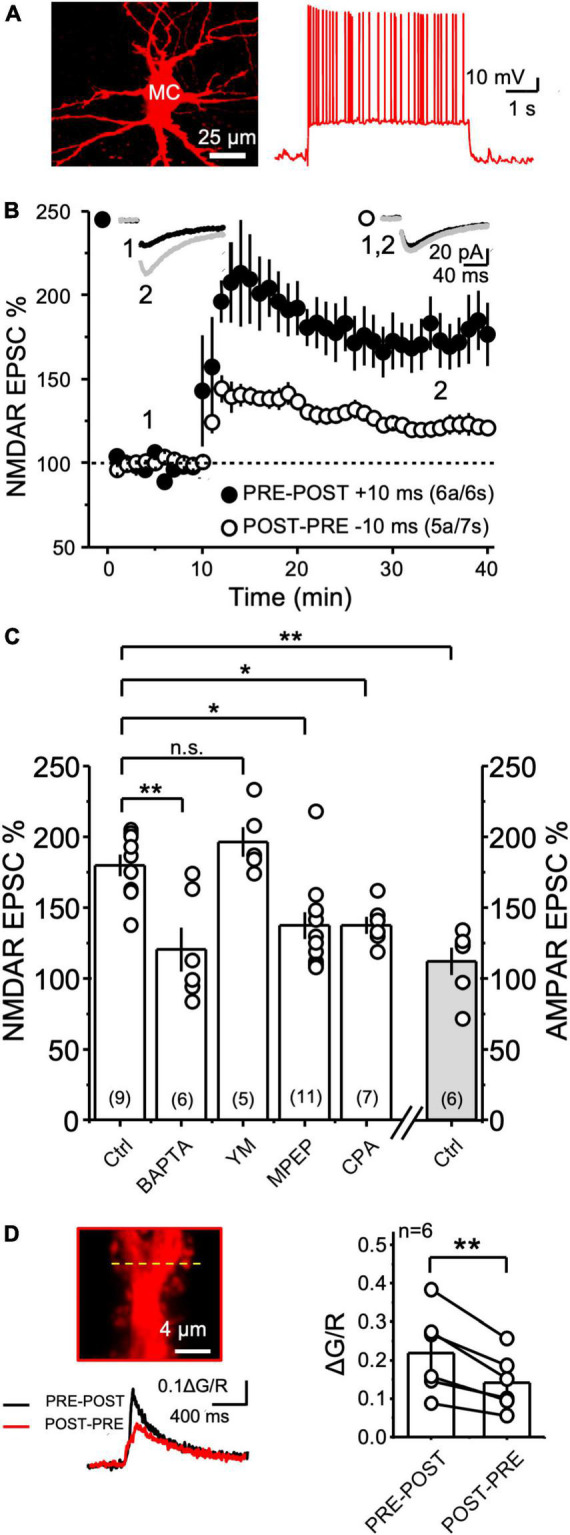
NMDAR plasticity at the MF-to-Hilar Mossy Cells synapse. **(A)** Representative 2P reconstruction of a MC patch-loaded with biocytin (left). MCs displayed regular action potential firing induced by current injection and minimal to no after hyperpolarization (right). **(B)** Both PRE-POST and POST-PRE pairing protocols induced NMDAR LTP but not LTD at MF-to-Hilar Mossy Cells (MF-MC) synapses. **(C)** MF-MC NMDAR LTP induced by PRE-POST pairing under different experimental conditions: control, 20 mM intracellular BAPTA, 1 μM YM and 4 μM MPEP, and 30 μM CPA. The PRE-POST pairing protocol had no effect on AMPAR-mediated synaptic transmission. One Way ANOVA; *F* = 9.19144; *p* = 0.0000161; DF = 4.; Ctrl vs BAPTA *p* = 0.00442; Ctrl vs YM *p* > 0.05; Ctrl vs MPEP *p* = 0.0243; Ctrl vs CPA *p* = 0.04. Bonferroni *post hoc* analysis. **(D)** Representative MC TE depicting the line scan (yellow line) where 2P calcium imaging was performed (top left), PRE-POST vs POST-PRE MF-MC calcium signals (bottom left), and summary plot (right). Data are presented as mean ± SEM. **p < 0.01, *p < 0.05, n.s. not significant.

MFs also make synapses with hilar mossy cells (MCs), and these synapses share structural and functional properties with MF-CA3 synapses, including the presence of giant MF boutons and postsynaptic TEs, a high degree of synaptic facilitation, regulation of glutamate release by mGluR2/3 agonists and the expression of presynaptic LTP (Lysetskiy et al., 2005; Scharfman, 2016). However, whether MF-MC synapses can undergo NMDAR plasticity was never tested. We therefore recorded MCs and delivered PRE-POST and POST-PRE parings like for MF-CA3 synapses. As previously reported, MCs in rat hippocampal slices were identified by the high frequency of spontaneous synaptic activity, regular action potential firing, and minimal-to-no afterhyperpolarization (Larimer and Strowbridge, 2008). In addition, MCs were patch-loaded with Alexa 594 and their identity was confirmed *post hoc* by the visualization of TEs (Figure 5A). We found that the PRE-POST pairing protocol induced robust NMDAR-LTP at MF-MC (Figure 5B, MC PRE-POST induction = 174 ± 14% of baseline; *n* = 6; *p* = 0.00075; paired t Test). In contrast, the POST-PRE protocol did not trigger NMDAR-LTD, but a small, yet significant, LTP (Figure 5B, MC POST-PRE induction = 121 ± 4% of baseline; *n* = 7; *p* = 0.0021; paired t Test). Importantly, the PRE-POST protocol did not induce LTP of the AMPAR-mediated component of MF-MC synaptic transmission (Figure 5C, AMPAR EPSC = 111 ± 9% of baseline, *n* = 6; Ctrl vs AMPAR *p* = 0.00089, One Way ANOVA). The selective strengthening of the NMDAR- but not AMPAR-mediated component supports a postsynaptic mechanism of NMDAR-LTP expression at MF-MC synapses.

To determine whether MF-MC NMDAR-LTP required postsynaptic calcium rise, we first loaded MCs with the calcium chelator BAPTA (20 mM). This manipulation significantly reduced the magnitude of NMDAR-LTP (Figure 5C, Control MC NMDAR LTP = 180 ± 7% of baseline, *n* = 9; NMDAR LTP + BAPTA = 120 ± 15% of baseline, *n* = 6; Ctrl vs BAPTA *p* = 0.00442, One Way ANOVA; *F* = 10.41736; *p* = 0.0000161; DF = 4; Bonferroni *post hoc*). Given the critical role of I-mGluRs for the induction of NMDAR-LTP at most central synapses (Hunt and Castillo, 2012), including the MF-CA3 synapse (Kwon and Castillo, 2008a; Rebola et al., 2008; Hunt et al., 2013), we also tested whether these receptors were implicated in MF-MC NMDAR-mediated plasticity. Bath application of the mGluR1 antagonist YM (1 μM) had no effect on NMDAR-LTP (Figure 5C, NMDAR LTP + YM = 196 ± 10% of baseline, *n* = 5; Ctrl vs YM *p* > 0.05, One Way ANOVA), whereas the mGluR5 antagonist MPEP (4 μM) significantly reduced the magnitude of NMDAR-LTP (Figure 5C, MPEP = 137 ± 9% of baseline, *n* = 11; Ctrl vs MPEP *p* = 0.0243, One Way ANOVA; Bonferroni *post hoc* analysis). To test whether downstream recruitment of calcium internal stores played a role in NMDAR-LTP at MF-MC synapse, we depleted calcium stores with CPA (30 μM). We found that this manipulation significantly reduced the magnitude of NMDAR-LTP (Figure 5C, CPA = 137 ± 5% of baseline, *n* = 7; Ctrl vs CPA *p* = 0.04, One Way ANOVA, Bonferroni *post hoc* test). Lastly, we tested the effect of MPEP and intracellular BAPTA on the small LTP induced by the POST-PRE protocol. While 4 μM MPEP had no effect, BAPTA blocked this plasticity (Ctrl POST-PRE LTP = 121 ± 4%, *n* = 7; BAPTA = 89 ± 15%, *n* = 4; MPEP = 140 ± 11%, *n* = 4; Ctrl POST-PRE LTP vs BAPTA *p* = 0.03; Ctrl POST-PRE-LTP vs MPEP *p* = 0.13; Mann Whitney test; data not shown). Altogether, these results strongly suggest that MF-MC synapses express a different learning rule for NMDAR plasticity compared with MF-CA3 synapses.

The expression of NMDAR-LTP and the lack of NMDAR-LTD at the MF-MC synapse indicate a functional difference between MF-CA3 and MF-MC synapses, which could rely on different postsynaptic calcium signals generated by the pairing protocols. To directly test this possibility, we measured CaTs at MF-MC TEs in response to PRE-POST or POST-PRE single pairings, as we performed at MF-CA3 synapses. To our surprise, PRE-POST and POST-PRE CaTs at MF-MC synapses resembled the pattern observed at MF-CA3 synapses (Figure 5D, PRE-POST = 0.22 ± 0.04; POST-PRE = 0.14 ± 0.03; PRE-POST vs POST-PRE: *n* = 6 *p* = 0.0045 paired t Test). Importantly, this difference was also preserved when analyzing single PRE-POST and POST-PRE trials (PRE-POST = 0.20 ± 0.05, *n* = 6; POST-PRE = 0.10 ± 0.01, *n* = 6; PRE-POST vs POST-PRE *p* = 0.03 Paired t Test, data not shown), discarding a potential priming effect. These findings indicated that other factors beyond postsynaptic calcium rise likely account for the different synaptic learning rule of MF-MC NMDAR plasticity.

### mGluR5 and mGluR1b distribution at MF-MC and MF-CA3 synapses differs

**FIGURE 6:**
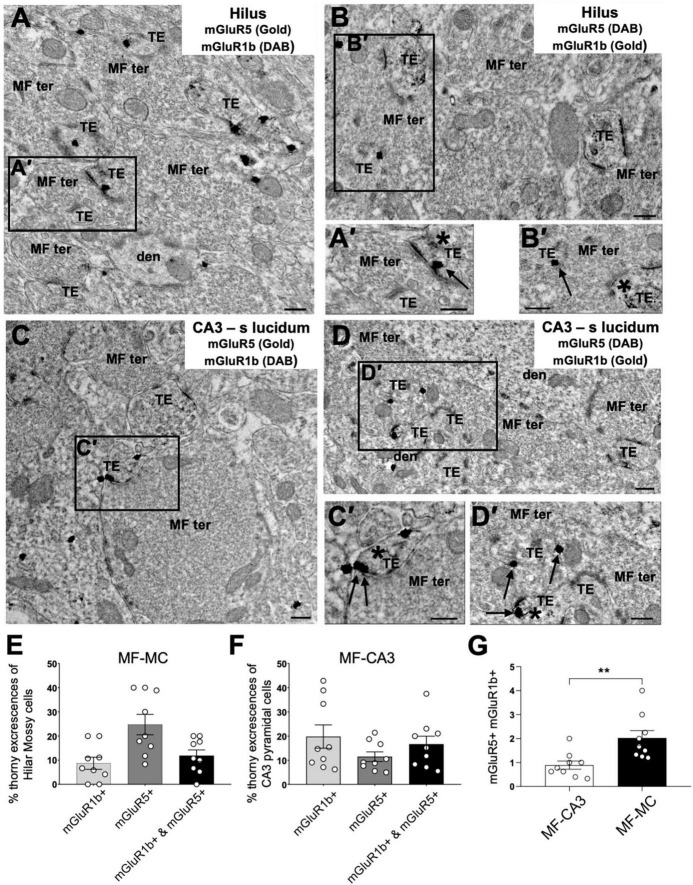
Immuno–electron microscopy of mGluR1b and mGluR5 at TEs in the hilus and CA3 stratum lucidum of hippocampus. **(A–D)** Large MF synaptic terminals (MF ter) containing abundant round and clear synaptic vesicles form asymmetric synapses with TEs in dendrites (den) of MCs in the hilus of the dentate gyrus **(A,B)** and CA3 pyramidal neurons **(C,D)**. MF synapses [insets **(A′,B′)**] contain mGluR1b [asterisk in panel **(A′)**; arrow in panel **(B′)**] and mGluR5 [arrow in panel **(A′)**; asterisk in panel **(B′)**] in dendritic TEs found on MCs. TEs of CA3 pyramidal neurons [insets **(C′,D′)**] also contain mGluR1b [asterisk in panel **(C′)**; arrow in panel **(D′)**] and mGluR5 [arrow in panel **(C′)**; asterisk in panel **(D′)**]. **(E,F)** Distribution of mGluR1b, mGluR5, or both mGluR1b and mGluR5 in TEs of MCs (*n* = 193) and CA3 pyramidal neurons (*n* = 158). **(G)** Ratio of mGluR5/mGluR1b at MF-CA3 and MF-MC synapses, demonstrating a distinct I-mGluR subtype expression at these synapses (**p = 0.0023). All data are expressed as mean ± SEM. Each data point in the bar graphs shows the mean value of the percentage **(E,F)** or the ratio **(G)** of each experiment from each animal. Scale bars: 0.25 μm.

MF-CA3 synapses mainly express mGluR5 and mGluR1b (Hunt et al., 2013), two receptors with distinct functional properties (Conn and Pin, 1997). For example, due to its short intracellular c-terminal tail, mGluR1b engages postsynaptic calcium rise from the intracellular calcium stores with less efficiency compared to mGluR5 and other mGluR1 splicing variants with longer c-terminal domain (Prezeau et al., 1996; Ohtani et al., 2014). To compare the expression of I-mGluR subtypes at MF-MC and MF-CA3 synapses side-by-side, we used double pre-embedding immunoelectron microscopy and determined the subcellular mGluR1b/mGluR5 distribution in the hilus (Figures 6A, B) and CA3 stratum lucidum (Figures 6C, D). Labeling for both receptors (asterisks and arrows in A**’**–D**’**) was observed within dendritic TEs forming asymmetrical synapses with typical MF terminals in both hippocampal subregions (Figures 6A–D). We found that MF-MC and MF-CA3 synapses displayed a different distribution of mGluR5 and mGluR1b, with MCs TEs expressing more mGluR5 and less mGluR1b than CA3 (Figure 6E; MC TEs with mGluR5: 24.7 ± 4.2%; MC TEs with mGluR1b: 8.7 ± 2.5%) (Figure 6F; CA3 TEs with mGluR5: 11.5 ± 2.0%; CA3 TEs with mGluR1b: 19.8 ± 4.3%). Remarkably, the proportion of mGluR5 versus mGluR1b, measured as the mGluR5/mGluR1b ratio, was significantly higher in MF-MC than MF-CA3 synapses (Figure 6G, MF-MC: 2.0 ± 0.3; MF-CA3: 0.9 ± 0.2, **p = 0.0023). Thus, consistent with the requirement of mGluR5 in NMDAR LTP and mGluR1 in NMDAR LTD previously reported at MF-CA3 synapses (Hunt et al., 2013), the different subcellular distribution of I-mGluR subtypes could account, at least in part, for the strikingly different NMDAR plasticity at these two similar synapses.

## Discussion

In this study, we report that the properties of NMDAR plasticity at MF synapses are target cell-specific. Specifically, we found that induction of NMDAR-LTP at the MF-CA3 synapse is associated with a larger postsynaptic calcium rise than the induction of NMDAR-LTD. This difference in calcium rise relied on mGluR5 activation and calcium release from internal stores, two key factors necessary for NMDAR-LTP induction, but not on mGluR1 activation. In contrast, the MF-MC synapse expressed robust NMDAR-LTP but no LTD. Surprisingly, despite the different learning rules, the calcium signals at MF-MC and MF-CA3 synapses were similar, suggesting that postsynaptic calcium rise alone cannot predict the sign of NMDAR plasticity. Lastly, we provide evidence for a different distribution of I-mGluRs at MF-MCs compared to MF-CA3 synapses, indicating that these receptors may play a critical role in controlling NMDAR plasticity.

### The role of postsynaptic calcium in bidirectional NMDAR plasticity

Previous studies have suggested that the direction of NMDAR plasticity is determined by the magnitude of postsynaptic calcium rise during induction. In midbrain dopamine neurons, burst timing-dependent NMDAR-LTP and LTD correlated with the levels of calcium in dendrites (Harnett et al., 2009), while in dentate granule cells, the direction of NMDAR plasticity was altered by the level of postsynaptic calcium buffering (Harney et al., 2006). In our study, by measuring postsynaptic calcium signals in dendritic spines with high spatiotemporal resolution, we determined that the order of the presynaptic and postsynaptic burst activity affects the magnitude of the postsynaptic calcium rise, which arises from multiple calcium sources. We found that NMDAR/mGluR5 coactivation is critical for the generation of larger CaTs during the PRE-POST protocol. Functional and structural interaction between mGluR5 and NMDAR has previously been described (Tu et al., 1999; Kotecha et al., 2003; Conn et al., 2009; Jin et al., 2013). Glutamate release from MF boutons likely co-activates NMDARs and mGluR5, leading to calcium release from the internal stores. Mechanistically, activation of NMDAR and mGluR5, as previously reported at MF-CA3 synapses (Kapur et al., 2001; Reid et al., 2001), could provide calcium influx and inositol 3 phosphate (IP3), respectively, two key requirements for IP3 receptors-mediated calcium release from the internal stores (Taylor and Laude, 2002; Cui et al., 2007). During the PRE-POST stimulation, the degree of coincidence detection by NMDAR is maximal due to the depolarizing effect of the backpropagating action potentials (i.e., POST stimulation) occurring while glutamate is still bound to NMDAR (Koester and Sakmann, 1998; Yuste et al., 1999; Feldman, 2012). Furthermore, PRE-POST patterns are associated with supralinear calcium summation, which relies on mGluR5 activation and calcium release from internal stores. Thus, it is possible that IP3 receptors act as coincidence detectors (Nakamura et al., 1999; Wang et al., 2000; Sarkisov and Wang, 2008) of robust elevations of calcium and IP3 triggered by NMDAR/mGluR5 coactivation. In contrast, reversing the order of bursts stimulation (i.e. POST-PRE) results in a sublinear calcium summation, which could be due to reduced NMDAR coincidence detection (Yuste et al., 1999; Shouval et al., 2002; Feldman, 2012), and calcium-dependent inactivation of NMDARs (Rosenmund et al., 1995; Tong et al., 1995). In addition to mGluR5, activation of voltage-gated calcium channels by backpropagating action potentials (i.e., POST) could also facilitate calcium release from the internal stores and contribute to the supralinearity. Thus, the high degree of NMDAR coincidence detection during the PRE-POST coupling together with mGluR5 coactivation could account for the supralinear calcium rise via activation of calcium release from the internal stores and likely constitutes a molecular switch for NMDAR-LTP.

Our findings support the long-standing theory developed for AMPAR-mediated synaptic transmission that intracellular calcium levels determine the direction of synaptic plasticity, i.e., larger postsynaptic calcium elevations are required for LTP and lower for LTD (Bear et al., 1987; Lisman, 1989; Artola and Singer, 1993). However, some specific points must be highlighted. We showed that blockade of mGluR5 and depletion of calcium stores, two manipulations that selectively block NMDAR-LTP at MF-CA3 synapses (Hunt et al., 2013), abolished the difference in postsynaptic calcium between the two induction protocols, lowering the PRE-POST CaTs to similar levels of POST-PRE CaTs. According to the two-threshold hypothesis (Bear et al., 1987; Lisman, 1989; Artola and Singer, 1993), this reduction is expected to convert NMDAR-LTP into LTD, which does not occur at MF-MC synapse. While these results do not exclude the existence of different calcium thresholds, it indicates that calcium levels are not the only determinant of NMDAR plasticity bidirectionality.

Previous work suggests that the direction of plasticity cannot be predicted by calcium levels alone but that additional molecular mechanisms occur in parallel to calcium elevations (Wang et al., 2005; Nevian and Sakmann, 2006; Piochon et al., 2016). It is important to acknowledge that local fast calcium signals (i.e., calcium nanodomains) that cannot be resolved with common imaging techniques, might also be required for the induction of plasticity. Other signaling pathways, parallel to I-mGluRs activation, might mediate a veto mechanism (Rubin et al., 2005) or gating mechanism that suppresses the generation of NMDAR-LTD regardless of the postsynaptic calcium levels. Consistent with this idea, AMPAR LTP or LTD in cultured hippocampal neurons does not seem to rely on postsynaptic calcium levels alone (Wang et al., 2005). Likewise, at the cerebellar parallel fibers-to-Purkinje cells (PF-PC) synapse, bidirectional AMPAR synaptic plasticity cannot be predicted by absolute calcium levels, but rather by relative calcium thresholds and by the presence of instructive signals for plasticity (Piochon et al., 2016). Future studies should determine the identity of a molecular veto mechanism for NMDAR-LTD at MF-CA3 and MF-MC synapses.

### Role of mGluRs in determining bidirectional NMDAR plasticity and functional implications

A critical component of bidirectional NMDAR plasticity at MF-CA3 synapses is the functional difference between mGluR5 and mGluR1 (Hunt et al., 2013). TEs express the mGluR1b splicing variant (Hunt et al., 2013), which lacks a portion of the intracellular C-terminal that enables high-efficiency activation of the Gq-coupled phospholipase C pathway (Prezeau et al., 1996). The short intracellular C-terminal is associated with reduced mGluR1b-mediated calcium release from internal stores (Ohtani et al., 2014), which is consistent with our observation that mGluR1 does not contribute to glutamate-evoked postsynaptic calcium rise (Figure 1C) and with previous reports of non-canonical, G-protein-independent mGluR1-mediated signaling in CA3 pyramidal neurons (Benquet et al., 2002; Gerber et al., 2007; Frausto et al., 2011). The precise mechanism by which mGluR1b controls NMDAR-LTD is unclear. NMDAR-LTD at MF-CA3 requires phosphatase activity (Hunt et al., 2013), but it is unknown whether mGluR1b is upstream regulators of neuronal phosphatases. mGluR1b can interact with cytoskeletal proteins that mediate mGluR-dependent regulation of dendritic spine structural dynamics (Francesconi et al., 2009; Kalinowska et al., 2015), a mechanism associated with other forms of structural LTD (Stein et al., 2015).

Changes in postsynaptic calcium dynamics due to NMDARs and/or mGluRs modulation could significantly affect the ability of synapses to undergo NMDAR plasticity. For example, calcium permeability of the NMDAR can be modulated by intracellular pathways that are typically downstream of GPCR activation (Skeberdis et al., 2006; Chalifoux and Carter, 2010; Higley and Sabatini, 2010; Murphy et al., 2014), raising the possibility that neuromodulatory projections, by regulating the amount of NMDAR-mediated calcium influx, could modify the threshold for NMDAR plasticity. Moreover, other G_*q*_-coupled receptors could act synergistically with mGluR5 (Cui et al., 2007; Tovar-Diaz et al., 2018) and bias the NMDAR plasticity threshold toward NMDAR-LTP. Because of the critical role of CA3 NMDARs in memory (Nakazawa et al., 2002, 2003; Nakashiba et al., 2009), such mechanisms could deeply impact hippocampal function.

### NMDAR plasticity at MF-MC synapses

While the calcium dynamics at MF-MC and MF-CA3 postsynaptic compartments was comparable, the former did not express NMDAR-LTD. Previous work indicates that mGluR1 is critical for NMDAR-LTD (Hunt et al., 2013; Bhouri et al., 2014). The lower expression of mGluR1b in MC TEs (Figure 6) could account for the lack of NMDAR-LTD at MF-MC synapses, although differences in calcium dynamics (Evans and Blackwell, 2015) and other molecular differences may also contribute. Even when compared to the already low NMDAR/AMPAR ratio of MF-CA3 synapses (Weisskopf and Nicoll, 1995; Hedrick et al., 2017), the MF-MC synapse expresses a remarkably low NMDAR/AMPAR ratio (Hedrick et al., 2017) and could therefore be prone to potentiation but not depression of NMDAR-mediated transmission.

MCs have been implicated in several hippocampal functions, such as spatial encoding and pattern separation (Danielson et al., 2017; GoodSmith et al., 2017; Senzai and Buzsaki, 2017) and novelty detection (Bernstein et al., 2019). MCs are also believed to play a critical role in neurological diseases such as epilepsy (Bui et al., 2018; Botterill et al., 2019). NMDAR plasticity at MF-MC synapses may contribute to the recruitment of MCs by GC activity and the encoding of multiple place fields, a key feature of MCs (Danielson et al., 2017; GoodSmith et al., 2017; Senzai and Buzsaki, 2017). Due to the slow kinetics of NMDAR-EPSCs, LTP of NMDAR-mediated transmission at MF-MC synapses may enable temporal summation of MF inputs during GCs bursting and facilitate GC-MC spike transfer. In addition, aberrant NMDAR plasticity could lead to excessive MCs firing and not only impair normal hippocampal function, but also promote runaway activity and epilepsy.

## Data availability statement

The original contributions presented in this study are included in the article/supplementary material, further inquiries can be directed to the corresponding author.

## Ethics statement

The animal study was reviewed and approved by Silvia E. Racedo, Ph.D., IACUC, Albert Einstein College of Medicine.

## Author contributions

SL, KA, and PC designed the experiments and wrote the manuscript. SL and KA performed the experiments and analyzed the 2P imaging and electrophysiology data. NP and PG generated the immunoelectron microscopy data. All authors edited the final version of the manuscript.
